# Effects of e-cigarette smoking on periodontal health: A scoping review

**DOI:** 10.1371/journal.pgph.0002311

**Published:** 2024-03-20

**Authors:** Priti Charde, Kamran Ali, Nader Hamdan

**Affiliations:** Qatar University, QU Health, College of Dental Medicine, Doha, Qatar; PLOS: Public Library of Science, UNITED STATES

## Abstract

**Objectives:**

Use of electronic cigarettes (ECs), also known as vaping, has gained remarkable popularity globally during the last decade especially among young people. Current evidence suggests that vaping may be associated with health risks. The aim of this study is to evaluate whether vaping increases the risk for initiation and progression of periodontal disease; and to appraise the clinical changes seen in patients using e-cigarettes, and how these changes impact the management of periodontal disease.

**Study selection, data and sources:**

A comprehensive electronic search was conducted on the PubMed, Scopus and Embase databases using the following search terms: Electronic Cigarettes OR vaping OR electronic nicotine delivery systems OR e-cigarettes AND Periodontitis. The search was limited to studies published from 1^st^ January 2012 to 31^st^ December 2022.

**Results:**

A total of 23 clinical studies focusing on the effect of e-cigarette smoking on the periodontal clinical parameters, levels of inflammatory mediators, alteration in periodontal microflora, and response to periodontal treatment were found to be eligible for inclusion in the review. Vaping may be associated with greater clinical attachment loss (CAL) compared to non-smokers. Moreover, ECs are also associated with unfavorable effects on periodontal microbial counts, biomarkers of inflammation and oxidative stress.

**Conclusions:**

Vaping may play a role in the initiation and progression of periodontal disease by altering the host response resulting in the release of inflammatory cytokines and periodontal microflora. Clinical studies show deleterious effects of vaping on periodontal health as well as less favourable response to periodontal treatment is observed in e–cigarette users compared to non-smokers. However, compared to cigarette smoking, the effects of vaping are less remarkable.

## Introduction

Electronic cigarettes (ECs) are nicotine delivery devices, referred to as e-cigarettes, electronic vaping devices, personal vaporizers, or vaping. They were created and copyrighted in Beijing, China, in 2003, and subsequently released in Europe and the United States in 2006 [1, 2]. The growing market of ECs has resulted in rapid evolution in their design, and available flavors and have the first generation was called "Cig-a-likes," the second "Vape pens," the third "Mods," and the fourth "Pod Mods" [3]. Over the last decade, ECs have been recommended as a smoking cessation aid by healthcare providers, especially to chronic smokers who wish to quit [4].

Although marketed as safer alternatives to traditional cigarettes, ECs contain several ingredients, which may be harmful and injurious to health. According to the food and drug administration (FDA) e-cigarette cartridges/solutions contain heavy metals, nitrosamines, diethylene glycol, and other potentially dangerous contaminants. The manufacturers claim that vape pen aerosols contain very low quantities of free radicals since vape pen do not burn tobacco. However, available evidence suggests that ECs may be associated with health risks and caution needs to be exercised before recommending ECs as a safe alternative to smoking cigarettes [5, 6].

Given the increase in popularity of vaping in recent years, particularly among young people, it is crucial to raise awareness that ECs are not free from risks to human health. Only 24% of current vapers understand the risks associated with using electronic cigarettes, while 74% of current smokers understand the risks associated with routinely smoking traditional cigarettes [7]. When ECs first emerged on the market, regulations regarding the manufacturing, sale and promotion of ECs were less stringent which allowed their rapid growth in the market. Only in the last 5 years, additional evidence regarding risks of ECs has mandated some countries to introduce tighter regulations to regulate the e-cigarette market. A single e-cigarette device may contain the same amount of nicotine as found in a pack of 20 traditional cigarettes [8]. Approximately 99% of the ECs sold in the U.S. contain nicotine, according to a recent CDC research; some labels do not state whether they do or not; and some of them do even though they are advertised as having 0% nicotine [9].

Recent investigations by the centers for Disease Control and Prevention, U.S. Food and Drug administration, and state health authorities in the United States have linked hospitalizations to the use of vaping products [10, 11]. An outbreak of acute and severe respiratory symptoms has compelled health agencies to conduct research on e-cigarette or vaping product use-related lung injury (EVALI). One of the primary causes of the EVALI outbreak is thought to be vitamin E acetate, which has been used as a diluent in vaping products. Another risk is blast injuries brought on by battery explosions, particularly in nations where e-cigarette manufacturing and safety remains unregulated [12].

Cigarette smoking has long been recognized as a risk factor for the development and progression of periodontal disease. However, the effects of vaping on periodontal health are not fully known. In vitro and in vivo studies have been conducted to assess the impact of vaping on periodontal tissue and the development and progression of periodontal disease. In vitro studies have shown that e-cigarette use alters myofibroblast differentiation, causes DNA damage, oxidative stress, and increases inflammatory cytokines in human gingival and periodontal ligament fibroblasts [13]. In a systematic review analyzing eight case-controlled studies, Figueredo et al. (2021) reported negative effect of vaping on periodontitis [7]. However, the authors reported that these effects were non-significant in the clinical context and results should be interpreted with caution due to a moderate risk of bias in four of the eight studies. Similarly a recent systematic review reported that compared to conventional tobacco smoking, ECs use may cause attenuated clinical inflammatory signs of periodontitis and, hypothetically, peri-implantitis [14]. Although in vitro studies have demonstrated negative effects of alternate tobacco products on periodontal health, analyses of clinical studies are required to assess the effect of vaping on periodontal health and its role in the pathogenesis of periodontal disease.

Periodontal disease is characterized by a progressive degeneration of tooth supporting soft and hard tissues, which is mediated by the interaction of dysbiotic microflora and aberrant immune responses in the gingival and periodontal tissues [15, 16]. Tobacco smoking is a recognized risk factor in the pathogenesis of periodontal disease and its role in progression of periodontal disease is well established [17, 18]. Tobacco smoking has been linked to exacerbated host immune response resulting in increased production of inflammatory mediators along with alteration in oral microflora [19]. Studies have demonstrated adverse effects of tobacco smoking on clinical and microbiological aspect of periodontal disease [20, 21]. The use of ECs is on rise in adults and young people [22, 23]. Yet, there is no clarity about how it affects the periodontium and periodontal health status particularly with respect to clinical changes. Recently, research has been carried out to evaluate impact of e-cigarette vaping on periodontal health, and changes in common clinical indicators of periodontal disease [7, 17]. Studies have also investigated levels of inflammatory mediators as well as, changes in periodontopathic microflora [21, 24, 25].

The aim of this review is to evaluate whether vaping increases the risk for initiation and progression of periodontal disease; and to appraise the clinical changes seen in patients smoking ECs, and how these changes impact the management of periodontal disease in patients who smoke ECs compared to smokers and non-smokers.

## Materials & methods

### Research question

How does vaping affect clinical periodontal parameters, inflammatory-mediator levels, periodontopathic microflora colonization in periodontal disease in comparison with conventional cigarette smoking?

Eligibility criteria for the studies were as follows:

Inclusion criteria:

- Type of study: clinical studies focusing on the effect of vaping on the periodontal clinical parameters, levels of inflammatory mediators, alteration in periodontal microflora, and response to periodontal treatment in human subjects;
- Period: Studies published from January 2012 to 31^st^ December 2022; and
- Language: Studies published in English were considered.

Exclusion criteria:

- Animal or cadaveric studies;
- In vitro studies;
- Studies conducted before 2012; and
- Studies published in languages other than English

### Search strategy

A comprehensive electronic search was conducted on the PubMed, Scopus and Embase databases using the following search terms: Electronic Cigarettes OR vaping OR electronic nicotine delivery systems OR e-cigarettes AND Periodontitis. The search was limited to studies published from 1^st^ January 2012 to 31^st^ December 2022.

**Fig 1:**
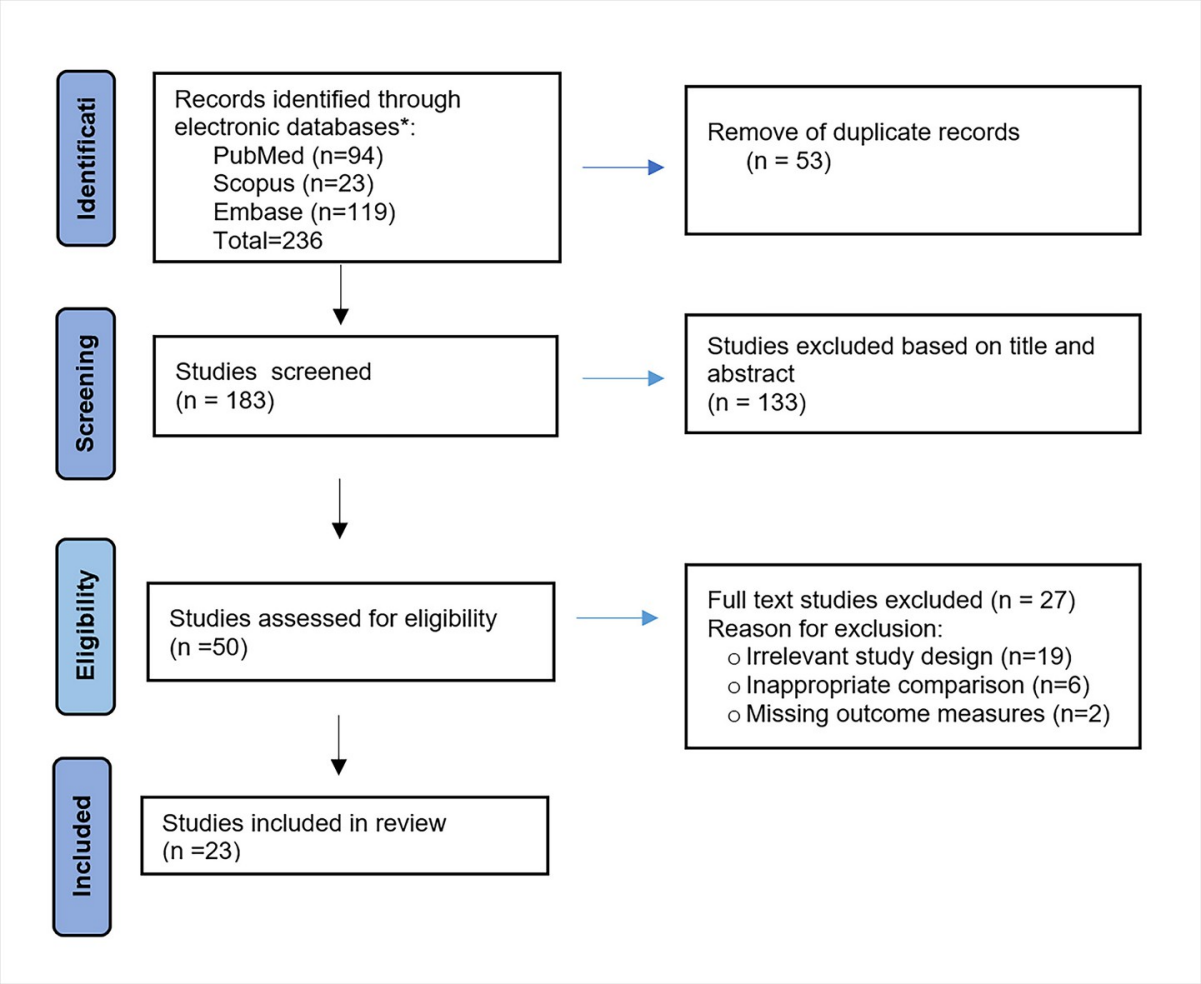
PRISMA flow chart.

The initial search identified a total of 236 records which were imported into EndNote, version 20.4.1 (Clarivate plc, London, UK), Clarivate Analytics. After the removal of duplicates (n = 53), title and abstract screening was done for all articles (n = 183)by two reviewers (PC and KA). A total of 50 studies were identified for full text screening. After removing studies with irrelevant study designs, inappropriate comparisons and missing outcome measures (n = 27), 23 clinical studies focusing on the effect of e-cigarette smoking on the periodontal clinical parameters, levels of inflammatory mediators, alteration in periodontal microflora, and response to periodontal treatment were found to be eligible for inclusion in the review (Fig 1). Any disagreements were resolved by consensus.

## Results

A total of 23 clinical studies focusing on the effect of e-cigarette smoking on the periodontal clinical parameters, levels of inflammatory mediators, alteration in periodontal microflora, and response to periodontal treatment were found to be eligible for inclusion in the review. These studies were grouped into three clusters based on what they reported, and the extracted data were reported as follows:

- Clinical parameters: (n = 14).
- Microbiology (n = 4).
- Treatment outcomes (n = 5).

### I. Clinical parameters

Table 1 summarizes the studies that evaluated the effects of vaping on clinical periodontal parameters compared to cigarette smokers and non-smokers. The studies focus on clinical periodontal parameters such as plaque index (PI), probing pocket depth (PPD), clinical attachment level (CAL), and marginal bone level (MBL). In two studies, along with periodontal parameters, authors evaluated self-reported parameters such as gingival bleeding, pain, and swelling in the gingivae [26–28]. Other studies assessed the levels of inflammatory mediators such as pro-inflammatory cytokines and oxidative stress markers in gingival crevicular fluid and/ or saliva [29–34].

**Table 1:** Effects of vaping on clinical parameters and inflammatory mediators of periodontal disease.

Author, Year, Title	Study design and Comparison Groups	Results
Mokeem et al. 2018 [20] Clinical and radiographic periodontal status and whole salivary cotinine, IL-1β and IL-6 levels in cigarette- and waterpipe-smokers and E-cig users	Cross-sectional study CS, water pipe-smokers (WS), E-cigs and NS	PI, Probing pocket depth (PPD), Clinical attachment level (CAL) and Marginal Bone Level (NBL) scores higher among CS &WS compared with E-cigs and NS. Whole salivary nicotine levels are similar all types of smokers; IL-1β & IL-6 levels higher in CS &WS.
Bin Shabaib et al. 2019 [24] Clinical periodontal status and gingival crevicular fluid cytokine profile among cigarette-smokers, electronic-cigarette users and never-smokers.	Cross-sectional study CS, E-cigs and NS	PI, CAL, MBL were poorer and GCF levels of proinflammatory cytokines concentrations of IL-1β, IL-6, IFN-γ, TNF-α and MMP-8 were higher in CS compared with E-cigs & NS.
Javed et al. 2017 [26] Comparison of Periodontal Parameters and Self-Perceived Oral Symptoms(OSs) Among Cigarette Smokers, Individuals Vaping Electronic Cigarettes, and Never-Smokers	Case -controlled study cigarette smokers (CSs), electronic cigarette smokers (E-cigs), and never-smokers (NS)	Periodontal inflammation including plaque index (PI) and Pocket depth ≥4 mm and self-perceived OSs were exacerbated in CSs compared with E-cigs and NS.
Karaaslan et al. 2020 [27] The effects of vaping electronic cigarettes on periodontitis	Case controlled study Tobacco-cigarettes(t-cig) smokers, E-cigs, Former Smokers	Both t-cigs and e-cigs had unfavorable effects on markers of oxidative stress and inflammatory cytokines (more marked with t-cigs), and smoking cessation has a beneficial effect.
Vohra et al. 2020 [28] Comparison of self-rated oral symptoms and periodontal status among cigarette smokers and individuals using electronic nicotine delivery systems	Case controlled study self-reported cigarette smokers and electronic nicotine delivery systems users (E-cigs and JUUL) and NS	Bad breath, Pain in teeth and gums are more frequently experienced by cigarette smokers than E-cigs and JUUL-users and NS. Cigarette smoking was associated with increased PI and PD than ENDS usage.
Alqahtani et al. 2020 [29] Electronic nicotine delivery system-induced alterations in oral health via saliva assessment	Cross sectional study Electronic-nicotine-delivery-systems (ENDS) users and NS	Salivary IL- b and TNF - α were increased ENDS than NS. IL-6 and IL-8 levels were similar in ENDS user and NS. Metabolite profiling determined that 368 salivary metabolites were expressed differently in ENDS users compared to NS.
Ye at al 2020 [30] Inflammatory biomarkers and growth factors in saliva and gingival crevicular fluid of e-cigarette users, cigarette smokers, and dual smokers: A pilot study	Cross sectional study CS,E-cigs, dual users (DS) and NS	The levels of salivary PGE2 and GCF inflammatory mediators were significantly higher in CS compared with NS, EC, and DS. Chronic E-cigs is associated with an adverse effect on periodontal health although to less extent than the effects of traditional cigarettes.
Ibraheem et al. 2020 [31] Comparison of RANKL and osteoprotegerin levels in the gingival crevicular fluid of young cigarette- and waterpipe-smokers and individuals using electronic nicotine delivery systems	Case controlled study CS, WS, (ENDS)-users and NS	The PI, PD, CAL, MBL and GCF (RANKL and OPG) were significantly higher in CS, WS, and ENDS users than NS. There was no significant difference in GCF RANKL and OPG levels among smokers.
Faridoun et al. 2020 [32] Salivary biomarker profiles in E-cigarette users and conventional smokers: A cross-sectional study	Cross sectional study E-cigs, CS, mixed smoker, and NS	PI and levels of IL-1β, IL-10, and IL-1RA were higher among e-cigarette users.
Ganesan et al. 2020 [33] Adverse effects of electronic cigarettes on the disease-naive oral microbiome	Cross sectional study CS, NS, E-cigs, former smokers currently using e-cigarettes, and concomitant cigarette and E-cigs.	E-cigs exert a powerful, detrimental effect on the subgingival ecosystem, altering the immunotolerance of the host. Levels of pro-inflammatory cytokines IL-2, IL-6, GM-CSF, TNF-α, and INF-γ were highest among CS followed by ES and NS. Level of anti-inflammatory cytokine IL-10 was lowest in CS followed by E-cigs and NS.
Ali et al. 2022 [34] Comparison of periodontal status and salivary IL-15 and -18 levels in cigarette-smokers and individuals using electronic nicotine delivery systems	Case Controlled study CS; ENDS-users; NS with periodontitis; and NS without periodontitis	PI, CAL, PD, and number of missing teeth were higher in cigarette smokers, ENDs and NS with periodontitis than NS without periodontitis. CS and END smokers had higher levels of IL-15 and IL-18 than NS with or without periodontitis. Among NS the levels of IL-15 and -18 were higher in those with periodontitis than those without periodontitis.
Akram et al. 2021 [35] Longitudinal evaluation of clinical, spectral and tissue degradation biomarkers in progression of periodontitis among cigarette and electronic cigarette smokers	Split mouth longitudinal study CS and E-cigs	A significantly increased attachment loss was observed at 6 months for both smokers when compared with baseline. MMP-8, CTX, smoking pack-years were significantly correlated with PD and CAL among both e-cigs and CS.
Jeong et al. 2020 [36] Associations of electronic and conventional cigarette use with periodontal disease in South Korean adults	Case controlled study E-cigs, CS, ex-users, and NS	Periodontal disease was more prevalent in E-cigs and CS than non-users (electronic cigarettes: odds ratio [OR] = 2.34, 95%confidence interval [CI] = 1.52 to 3.59, conventional cigarettes: OR = 2.17, 95%CI = 1.76 to 2.68) than NS.
Xu et al. 2021 [37] Comparative Effects of e-cigarette Aerosol on Periodontium of Periodontitis Patients	Longitudinal study E-cigs, CS and NS	Carbon monoxide and salivary cotinine levels were highest among CS. BOP and average PPDs similarly increased over time in all three groups, but CAL uniquely increased in E-cigs. Rates of severe periodontal disease were higher in both CS and E-cigs users than NS, but interpretation is confounded by the older age of the CS. E-cigs had an increased risk of CAL progression.

Among studies evaluating the effect of vaping on clinical parameters and inflammatory mediators levels, Bin Shabaib et al. (2019) compared the clinical parameters such as plaque index, bleeding on probing, probing pocket depth, clinical attachment level, marginal bone loss and number of missing teeth and GCF inflammatory mediators levels among cigarette smokers, vapers and non-smokers [24]. Authors reported poorer periodontal status and levels of inflammatory mediators among conventional smokers compared to vapers and non-smokers. Karaaslan (2020) reported that CS and ECs had the same unfavorable effects on the markers of oxidative stress and inflammatory cytokines [27]. Similarly, Ibraheem et al. (2020) reported increased expression of RANKL in the GCF of e-cigarette smokers, similar to cigarettes and water pipe smokers, along with worsening of periodontal inflammation [31]. Similar observations were reported in other studies which showed that conventional smoking has more deleterious effects on periodontal clinical parameters including plaque index, probing pocket depth, clinical attachment level, and marginal bone levels and levels of inflammatory mediators such as IL-1β, IL-2, IL-6, IFN-γ, TNF-α, PG-2 and MMP-8 CAL compared to vapers and non-smokers [21, 26, 28, 32, 33, 36]. However, when compared with non-smokers increased inflammation and levels of inflammatory mediators were reported in electronic-cigarette users.

However, Xu et al. (2021) reported greater CAL loss in e-cigarette smokers [M (SD) = 2.9 (1.5) mm] and cigarette smokers [M (SD) = 3.5 (1.1) mm] than in the non-smokers [M (SD) = 2.2 (0.8) mm] [37]. With time, the increase of about 2 mm in CAL in the e-cigarette smokers was greater than that seen in the non-smokers or the cigarette smokers, indicating adverse effect of vaping on CAL parameter. But there were limitations in the study design due to lack of control over variables such as age and race, and the follow-up interval was short. In addition, variations in the type and brands of e-cigarette, e-liquid components, flavoring agents, and voltages should be considered while interpreting the results.

### II. Microbiology

Table 2 summarizes studies that evaluated how vaping affects periodontal bacterial composition in subgingival plaque samples when compared to samples collected from cigarette smokers and non–smokers. In two studies, along with alteration in microbial composition, authors evaluated the adverse effects on the levels of inflammatory biomarkers [38, 39]. Among these studies, Thomas et al. (2022) reported that most severe cases of periodontitis in conventional smoker group, mild cases in non-smoker group and presence of balanced number of all type of cases in vapers [40]. The oral microbial composition of heavy vapers was reported to be similar to that of heavy cigarette smokers but different than non-smokers of same stage of periodontitis. In addition to this, Aldakheel et al. (2020) and Pushalkar et al. (2020) reported a shift in more pathogenic microflora amongst vapers [25, 38]. Notwithstanding the limitations of these studies, vaping alters the periodontal microbiome, with growth of microflora seen in both conventional cigarette smokers and nonsmokers

**Table 2:** Effects of vaping on alterations in periodontal microflora.

Aldakheel et al. 2020 [25] Quantification of pathogenic bacteria in the subgingival oral biofilm samples collected from cigarette-smokers, individuals using electronic nicotine delivery systems and non-smokers with and without periodontitis	Case controlled study CS and ENDS-users with periodontitis, compared to NS with and without periodontitis.	Colony forming unit (CFU) /mL *Aggregatibacter actinomycetemcomitans*, *Porphyromonas gingivalis* and *Treponema denticola* were significantly higher among CS and ENDS-users than NS with periodontitis compared with NS without periodontitis.
Pushalkar et al. 2020 [38] Electronic Cigarette Aerosol Modulates the Oral Microbiome and Increases Risk of Infection	Cross sectional study E- cigs, CS and NS	Abundance of *Porphyromonas* and *Veillonella* (p = 0.008) was higher among vapers. IL-6 and IL-1β were highly elevated in e-Cigs when compared with NS.
Xu et al. 2022 [39] Electronic cigarette use enriches periodontal pathogens	Longitudinal study CS, E-cigs and NS	CS and E-cigs shared more similarities in their oral bacterial composition for patients with same stage of periodontal disease. E-cigs was also associated with elevated levels of proinflammatory cytokines such as IFN-γ and TNF-α, which contribute to oral microbiome dysbiosis and advanced disease state similar CS.
Thomas et al. 2022 [40] Electronic Cigarette Use Promotes a Unique Periodontal Microbiome.	Longitudinal study E-cigs, CS and NS	E -cigarette use may promote a healthier subgingival periodontal microbiome with respect to that of CS but not compared to that found with never smoking in the first place.

### III. Treatment outcomes

Table 3 summarizes the findings of studies focused on periodontal treatment outcomes in vapers compared to cigarette smokers and non-smokers. Studies compared changes in PI, gingival index (GI), PPD and CAL in the separate groups. Among these studies, only one randomized controlled trial evaluated the use of non-surgical periodontal therapy with adjunct use of photodynamic therapy for the management of periodontal inflammation in vapers and compared it with non-surgical periodontal treatment alone [41].

**Table 3:** Studies focusing on the effects of periodontal therapy on periodontal inflammation in e-cigarette smokers.

Author and Year, Title	Study design and Groups	Results
Alshibani et al. 2022 [41] Non-surgical periodontal therapy with adjunct photodynamic therapy for the management of periodontal inflammation in adults using nicotine-free electronic-cigarette: A randomized control trial	Randomized controlled trial 46 Patients randomly divided into test (Non-Surgical Periodontal Therapy + PDT) and control groups (Non-Surgical Periodontal Therapy) alone	Significant reduction in PI, BI and PPD in test and control groups at 3 months follow-up compared with baseline scores. No significant difference between groups.
Shah et al. 2022 [42] Retrospective exploratory study of smoking status and e-cigarette use with response to non-surgical periodontal therapy	Retrospective study 220 patients with periodontitis: former smokers (n = 60); former smokers now using E-cigs (n = 20); current smokers (n = 20), non-smokers (n = 120).	Compared with NS, E-Cigs had a less favorable treatment response after professional mechanical plaque removal. No significant differences between groups. There was a statistically significantly better response by former smokers compared to E-Cigs.
Alhumaidan et al. 2022 [43] Comparison of Whole Salivary Cortisol and Interleukin 1-Beta Levels in Light Cigarette-Smokers and Users of Electronic Nicotine Delivery Systems before and after Non-Surgical Periodontal Therapy	Quasi Experimental study CS (n = 18), ENDS users (n = 18) and NS (n = 18)	At 12-weeks of follow-up, PI and PD were significantly high in CS and ENDS-users compared to NS. In NS, significant reduction in IL-1β and CL at 12 weeks of follow-up compared with baseline. In CS and ENDS users, clinical periodontal parameters and whole-salivary CL and Il-1β levels remain unchanged after NSPT.
Al Hamoudi et al. 202 [44] Effect of scaling and root planning on the expression of anti-inflammatory cytokines (IL-4, IL-9, IL-10, and IL-13) in the gingival crevicular fluid of electronic cigarette users and non-smokers with moderate chronic periodontitis	Cross sectional study E-cigs (n = 30) and NS (n = 35) with moderate chronic periodontitis (CP).	At the 3-month follow-up, there were no significant differences in PI, gingival index (GI), PD, CAL, and MBL in E-cigs while there were significant reductions in PI, GI, and PD among NS compared to baseline. GCF IL-4, IL-9, IL-10, and IL-13 levels were significantly elevated in both groups.
AL Harthi et al. 2019 [45] Impact of cigarette smoking and vaping on the outcome of full-mouth ultrasonic scaling among patients with gingival inflammation: a prospective study	Prospective study CS (n = 30), E-cigs (n = 28), and NS (n = 31)	At follow-up visits, in group 1, there was no statistically significant difference in mean PI and PPD and numbers of sites with PD ≥ 4 mm. In groups 2 and 3, there is no significant difference in PI, BOP, and PPD. However, there were no pockets with PD ≥ 4 mm at 3- and 6-months follow-up in groups 2 and 3.

Less favorable treatment response was reported in vapers showing that more patients need surgery after professional mechanical plaque removal along with an increased number of sextants with PD ≥5 mm, number of sites with PD >5 mm and mean PD [42]. Compared to cigarette smokers, less favorable response to periodontal treatment in vapers was also reported by Alhumaidan et al. 2022 [43] and Al Hamoudi et al. 2020 [44]. However, due to the limitations in study design and a small sample size in these studies, results should be interpreted with caution.

## Discussion

Several studies have explored whether vaping increases the risk for initiation and progression of periodontal disease and attempted to quantify changes seen in vapers. Other studies have evaluated the impact of vaping on the management of periodontal disease compared with conventional cigarette smokers and non-smokers. Results of the studies focusing on changes in clinical periodontal parameters, inflammatory mediators and periodontopathic microflora suggest the following three mechanisms regarding how vaping affects the periodontium: impaired immune and inflammatory response, a shift in micro flora favoring periodontal pathogens, and subsequent changes in the healing capacity of soft tissue [24, 39, 44]. These mechanisms are similar to the deleterious effects of conventional smoking. Studies have demonstrated that pathogenic bacteria in samples of vapers were high when compared with non-smokers suggesting vaping causes oral environmental shifts and influences the colonization of complex heterogeneous microbial biofilms [25, 38–40]. Levels of inflammatory mediator both in GCF and saliva including interleukin-1β, IL-6, TNF- α, IL-4 and IL-8 found elevated in vapers when compared with nonsmokers while levels of anti-inflammatory mediators were low [30–32, 34, 35].

Patients with periodontal inflammation showed increased scores of plaque index (PI), bleeding index (BI), and probing depth (PD) and clinical attachment loss (CAL) which If left untreated, results in loss of teeth [41–43]. Attempts to intervene and inhibit an exuberant inflammatory response traditionally includes treatment modalities targeting removal of microbial etiology of periodontal disease. However, with better understanding of role of host response in pathogenesis of periodontal disease, there has been a shift towards host modulation along with traditional mechanical therapy. Clinical studies indicate exacerbation of host response and change in periodontopathic microflora in e-cig smokers similar to conventional smokers [46, 47].

Studies have been carried out to evaluate treatments to eliminate periodontal inflammation in vapers. When the periodontal treatment needs of e-cig smokers were compared with conventional smokers, conventional smokers needed more complex periodontal treatment compared to vapers [48]. It was observed that most of the cigarette smokers had pockets ≥6 mm (75%) while the majority of the vapers had calculus deposits (50%). On the other hand, 90% of the non-smokers had healthy periodontal tissues. Majority of studies has evaluated the effect of non-surgical periodontal therapy on gingival inflammation. Alshibani et al., (2022) used non-surgical periodontal therapy with adjunct photodynamic therapy for the management of periodontal inflammation [41]. Results of these studies suggested that gingival inflammation is worse in cigarette smokers compared with vapers and nonsmokers and levels of anti-inflammatory markers is low following non-surgical periodontal therapy indicating poor response to treatment.

Multiple factors which might affect the outcome of these studies including the types of vaping devices generation and design, amount of power wattage, temperature delivered to the system, composition of e-liquids (particularly types of flavoring and concentration of nicotine used), time and frequency of inhalation [49]. Most available studies investigating the effects of vaping on periodontal health are based on cross-sectional study designs which do not allow establishing a causal relationship nor can the association be interpreted reliably [50]. Nevertheless, ECs may have a detrimental effect on periodontal health, though the nature and magnitude of such effects requires further evidence. From a methodological perspective, large scale, multi-centered, randomized controlled trials would be ideal to investigate the effects of vaping on the periodontium. However, ethical concerns preclude such an approach due to known risks associated with smoking. Nevertheless, it is important to control confounding variables and record demographic information of participants comprehensively [51].

Notwithstanding the effects of ECs on periodontal and oral health, there is growing evidence regarding serious health risks with vaping. ECs have been marketed as a “safer” alternative to cigarette smoking, which has encouraged a remarkable trend in their use worldwide, especially amongst younger people. However, vaping is associated with risks to pulmonary and cardiovascular health. Vaping liquids are known to contain multiple definite and potential carcinogens [52]. Moreover, development of cancer in cigarette smokers from smokers indicates lag times of 2–3 decades before cancer development. Given that ECs have only been used on a wide scale in 15–18 years, precise estimation of their carcinogenic risk requires more time [53]. Vaping is also reported to increase the risk of acute toxicity, but risks of long-term toxicity are unknown [54]. The risk of acute lung injury with vaping has been reported widely [22, 55]. It has also been reported that in young adults with history of former or current cigarette use, switching from combustible cigarettes to vaping does not confer reduce the odds of having a stroke [56]. The higher prevalence of e-cigarette use amongst younger people is a particular concern and more stringent anti-vaping legislation is likely in future to address this issue.

A recent Cochrane review has reported that ECs with nicotine increase quit rates when compared to nicotine replacement therapy and ECs without nicotine [57]. Results of a systematic review and meta-analysis show that the odds of quitting cigarettes were 28% lower in vapers compared with non-vapers (odds ratio [OR] 0·72, 95% CI 0·57–0·91) [58]. Given the uncertainties about the risks of long-term vaping and its potential role as a gateway into smoking, particularly among young people, healthcare workers need to exercise caution when recommending vaping as a smoking cessation strategy. Also, future evidence about the harmful effects of vaping may prompt medico-legal litigation against healthcare providers. Based on the available evidence, the health risks of vaping need close monitoring. Nonsmokers and adolescents should be actively discouraged from vaping. Smokers should also be discouraged from engaging in dual use without cigarette reduction or cessation [59].

Our findings are in accord with previous systematic reviews which have reported that vaping is associated with adverse effects on periodontal health, although the severity of periodontal damage is less compared to cigarette smoking [7, 57]. In addition to the effects on periodontal health, ECs are reported to be associated with a wide range of oral health sequelae such as cough, throat and mouth irritation, and intraoral lesions such as nicotinic stomatitis, hairy tongue, and hyperplastic candidiasis [60]. In addition, flavored e-liquids may be associated with an increased risk of dental caries and the presence of inflammatory and carcinogenic biomarkers in saliva and crevicular fluid [61]. The current review underscores the need for consistent advice by dentists regarding the use of ECs as a smoking cessation option. Although further evidence is required for accurate quantification and long-term health risks of vaping, it is clear that e-cigarettes are not free from adverse effects on oral and systemic health and therefore, should not be considered as a “harmless alternative” to cigarette smoking.

There is a growing use of ECs as an alternative to cigarettes and the global market of ECs is forecast to increase from 22.17 billion USD in 2022 to 168.96 billion USD by 2030 which equates to a compound annual growth rate (CAGR) of 28.9% [62]. These market trends indicate that dentists will continue to encounter patients ECs. There are contradictions in the guidelines provided by leading healthcare bodies regarding the use of ECs. For example, the national health service (NHS) in the UK considers ECS to be suitable for smoking cessation including pregnant women [63]. On the other hand, the US Food and Drugs Administration (FDA) has not approved ECs for smoking cessation and the advice from the Centers for Disease Control and Prevention (CDC) USA states that pregnant women must avoid it completely [64]. Similarly, the World Health Organization (WHO), considers ECs to be harmful not only for the primary users but may harm people who are exposed to second hand vapors [65]. In contrast, the advice on the NHS stop- smoking website states that there is no evidence that second hand vape aerosol is harmful. Given the scale of vaping globally, it is important that the relevant international bodies work together to achieve a more common ground on ECs and support healthcare professionals including dentists to provide coherent advice to the public.

## Conclusion

Vaping plays a role in the initiation and progression of periodontal disease by altering the host response resulting in the release of inflammatory cytokines and periodontal microflora. Although the effects are less remarkable, vaping may be associated with adverse effects on periodontal health. Less favourable response to periodontal treatment has been observed noted in vapers compared to non-smokers. Further well-designed studies involving larger samples may help to reliably quantify the risks of vaping on periodontal health and inform professional guidelines to help dentists provide evidence-based advice on vaping to the public.
